# Pregnancy related complications in women with hypertrophic cardiomyopathy: a nationwide population-based cohort study

**DOI:** 10.1186/s12872-024-03812-3

**Published:** 2024-05-21

**Authors:** Won Yeol Choi, Kyung-Taek Park, Hyue Mee Kim, Jun Hwan Cho, Gina Nam, Joonhwa Hong, Dongwoo Kang, Jungkuk Lee

**Affiliations:** 1 Division of Cardiology, Department of Internal Medicine, Chung-Ang University Hospital, Chung-Ang University College of Medicine, 84 Heukseok ro, Dongjak-gu, Seoul, 06974 South Korea; 2 Heart and Brain Hospital, Chung-Ang University Gwangmyeong Hospital, Chung-Ang University College of Medicine, Gyeonggi, South Korea; 3 Department of Obstetrics and Gynecology, Chung-Ang University Hospital, Chung-Ang University College of Medicine, Seoul, Korea; 4 Department of Thoracic and Cardiovascular Surgery, Chung-Ang University Hospital, Chung- Ang University College of Medicine, Seoul, South Korea; 5 Data Science Team, Hanmi Pharm. Co., Ltd, Seoul, Korea

**Keywords:** Hypertrophic cardiomyopathy, Maternal outcomes, Pregnancy

## Abstract

**Background:**

The impact of hypertrophic cardiomyopathy (HCM) on cardiovascular and obstetrical outcomes in pregnant women remains unclear, particularly in Asian populations. This study aimed to evaluate the maternal cardiovascular and obstetrical outcomes in Korean women with HCM.

**Methods:**

Using data from the Korean National Health Insurance Service database, we identified women who gave birth via cesarean section or vaginal delivery after being diagnosed with HCM between 2006 and 2019. Maternal cardiovascular and obstetrical outcomes were assessed based on the trimester of pregnancy.

**Results:**

This study included 122 women and 158 pregnancies. No maternal deaths were noted; however, 21 cardiovascular events, such as hospital admission for cardiac problems, including heart failure and atrial fibrillation (AF), new-onset AF or ventricular tachycardia (VT) occurred in 14 pregnancies (8.8%). Cardiac events occurred throughout pregnancy with a higher occurrence in the third trimester. Cesarean sections were performed in 49.3% of the cases, and all cardiovascular outcomes occurring after delivery were observed in patients who had undergone cesarean sections. Seven cases involved preterm delivery, and two of these cases were accompanied by cardiac events, specifically AF. Pre-existing arrhythmia (AF: odds ratio (OR): 7.44, 95% confidence interval (CI): 2.61–21.21, *P* < 0.001; VT: OR: 31.61, 95% CI: 5.85–172.77, *P* < 0.001) was identified as a predictor for composite outcomes of cardiovascular events or preterm delivery.

**Conclusions:**

Most pregnant women with HCM were well-tolerated. However, cardiovascular complications could occur in some patients. Therefore, planned delivery may be necessary for selected patients, especially the women with pre-existing arrhythmias.

**Supplementary Information:**

The online version contains supplementary material available at 10.1186/s12872-024-03812-3.

## Introduction

Hypertrophic cardiomyopathy (HCM) is one of the most prevalent genetic diseases which can lead to adverse cardiovascular outcomes, including heart failure (HF), atrial fibrillation (AF), thrombosis and sudden cardiac death [1]. With the increased use of echocardiography and genetic screening, the incidence of HCM diagnosis has gradually increased. A recent study from a team of South Korean researchers demonstrated a prevalence of approximately 0.031% in patients with a mean age of > 60 years. Notably, the number of patients with HCM is also increasing among individuals in their 20 and 30 s, as well as among women, albeit at a slower rate [2]. 

Pregnancy induces various hemodynamic changes, such as decreased peripheral vascular resistance, increased cardiac output and tachycardia. During the gestational period, the left ventricular chamber undergoes dilation attributed to increased blood volume and enhanced contractility, while tachycardia concurrently reduces diastolic filling time [3, 4]. Consequently, HF may be more pronounced in patients with HCM during pregnancy. Additionally, vaginal delivery can cause a significant increase in heart rate and cardiac output, [5] while general or spinal anesthesia for cesarean section can reduce systemic vascular resistance and blood pressure owing to the parasympathetic effect [6]. Theoretically, these hemodynamic changes during delivery could provoke adverse events in patients with HCM. However, the actual impact of pregnancy in patients with HCM remains inadequately understood because of the small sample size [7–10]. Despite being at a relatively higher risk for cardiovascular issues, most pregnant patients with HCM tolerated their pregnancies well. However, reported complication rates varied widely, ranging from 15 to 45%, depending on the reports [7–10]. Therefore, this study aimed to evaluate maternal outcomes during pregnancy in patients with HCM using the Korean National Health Insurance Service database.

## Methods

### Data sources

This study was based on a database provided by the National Health Insurance Services-Health Screening (NHIS-HEALS) cohort in Korea. The NHIS is a mandatory insurance service in Korea covering 97.2% of the Korean enrollees, with individuals aged ≥ 40 being eligible for a general health screening program every 2 years. The remaining enrollees with a low income are covered by the Medical Aid Program, and the information has been incorporated into a single database since 2006. The database includes data regarding demographics, inpatient and outpatient services utilization, diagnoses, prescriptions, deaths and health screening examinations. The cohort details have been described previously. The study was approved by the Institutional Review Board of Chung-Ang University Hospital (#2108-011-19379). The anonymized dataset and unidentified information were provided to the researchers from the NHIS, and the requirement for informed consent was waived.

### Definition of HCM and pregnancy

HCM was defined by (1) claims for diagnostic codes (International Classification of Disease 10th Revision, ICD-10, I42.1 or I42.2) with at least one admission or outpatient visit, and (2) registration in the Korean Rare Intractable Disease (RID) program (code: V127), which includes patients with HCM. A previous study from Korea validated the definition using hospital data and showed high accuracy [11]. Pregnant patients were identified among the patients diagnosed with HCM according to the above definitions. Given the nature of the data, determining the precise onset of each patient’s pregnancy was not possible. Therefore, we utilized codes for vaginal delivery and cesarean section as indicators of pregnancy. The definitions of vaginal delivery and cesarean section were based on ICD-10 (Supplementary Table 1).

### Definition of comorbidities and pregnancy trimester

Comorbidities, including hypertension, diabetes, dyslipidemia, stroke, AF, HF and ventricular tachycardia (VT), were defined using the ICD-10 code and the prescription list in the NHIS database. The detailed definition of comorbidities is summarized in Supplementary Table 1. In pregnancy, the first day of the last menstrual period (LMP) was defined as the beginning of the first trimester (“day zero”). However, LMP was unavailable in the NHIS data, and the algorithm assumed “day zero” as the delivery date minus 39 weeks (273 days), if there was no code for preterm birth. If there was a code for preterm delivery (O601, O603), day zero was assumed as the delivery date minus 35 weeks (245) days [12, 13]. Therefore, baseline comorbidities were only included if diagnosed before 39 weeks prior to the delivery date (or 35 weeks for preterm delivery). The detailed definition of trimesters is summarized in Supplementary Table 2. We collected prescriptions for medications related to renin-angiotensin-aldosterone system (RAS) blockers, beta-blockers, calcium channel blockers, and amiodarone that were prescribed in Korea up to the estimated first day of pregnancy (Table 1).

**Table 1:** Baseline characteristics

Variables	Total (*N* = 158)	Cesarean Section (*N* = 78)	Vaginal delivery (*N* = 80)	*P*
Age	34.1 ± 4.3	35.2 ± 4.0	33.1 ± 4.2	0.002
Nullipara	122 (77.2%)	63 (80.8%)	59 (73.8%)	0.293
ICD implantation	0 (0%)	0 (0%)	0 (0%)	
Septal myectomy	2 (1.3%)	2 (2.6%)	0 (0%)	0.150
**Comorbidities**				
Hypertension	69 (43.7%)	31 (39.7%)	38 (47.5%)	0.326
Diabetes mellitus	3 (1.9%)	3 (3.8%)	0 (0%)	0.077
Stroke	0 (0%)	0 (0%)	0 (0%)	
Atrial fibrillation	24 (15.2%)	10 (12.8%)	14 (17.5%)	0.413
Heart failure	26 (16.5%)	15 (19.2%)	11 (13.8%)	0.353
Smoking	11 (7.0%)	5 (6.4%)	6 (7.5%)	0.789
Ventricular tachycardia	8 (5.1%)	3 (3.8%)	5 (6.3%)	0.491
**Medications before pregnancy**				
Beta blocker	72 (45.6%)	38 (48.7%)	34 (42.5%)	0.433
Calcium channel blocker	29 (18.4%)	15 (19.2%)	14 (17.5%)	0.779
Amiodarone	5 (3.2%)	4 (5.1%)	1 (1.3%)	0.164
RAS blocker	29 (18.4%)	20 (25.6%)	9 (11.3%)	0.020

Abbreviations: ICD, implantable cardioverter-defibrillator; RAS, renin-angiotensin-system

### Definition of cardiovascular and obstetrical outcomes

We evaluated cardiovascular and obstetrical outcomes during pregnancy and one month after delivery. Hospitalization due to HF and AF, thromboembolic events, new-onset AF and new-onset VT were evaluated as cardiovascular events. The detailed definitions of outcomes are summarized in Supplementary Table 1. In cases where both new-onset AF and AF-related hospitalization were recorded within the same specification, the latter was included, and the former was excluded from the analysis. Preeclampsia, gestational hypertension, placenta previa, intrauterine growth retardation and preterm delivery were evaluated as obstetrical outcomes.

### Statistical analysis

Categorical variables were presented as percentages, and continuous variables as means ± standard deviations. Patient characteristics between groups were compared using Pearson’s chi-squared test for categorical variables and Student’s t-test for continuous variables. Logistic regression analysis was performed to identify independent variables associated with adverse outcomes in pregnancy. To investigate the correlation in detail, factors considered as risk factors in pregnant HCM patients were analyzed. A two-sided significance level of *P* < 0.05 was considered statistically significant. Statistical analyses were performed using the SAS software (version 9.4; SAS Institute, Cary, NC, USA).

## Results

### Baseline characteristics

**Fig. 1:**
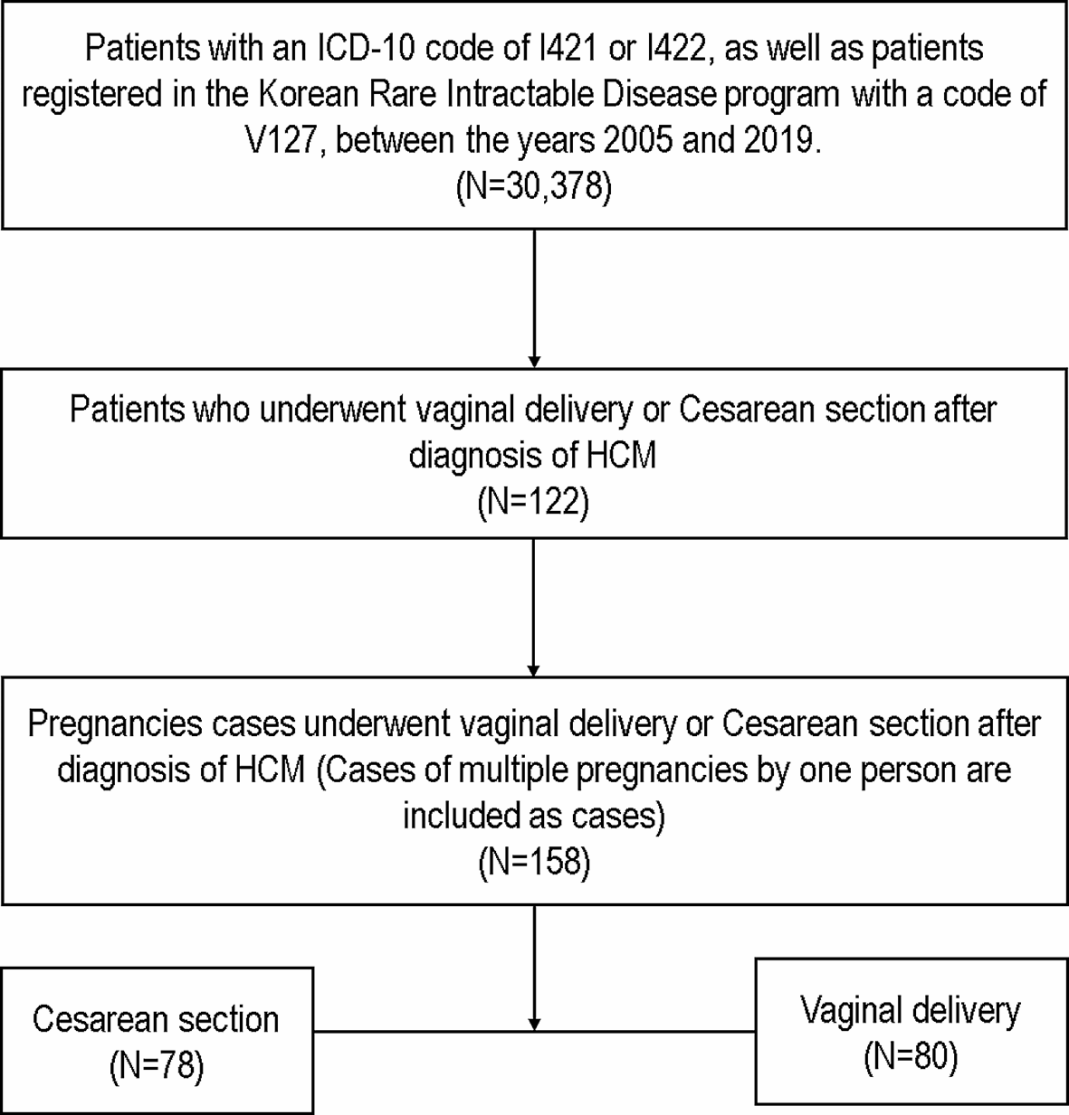
Flow chart of the study population Abbreviations: HCM, hypertrophic cardiomyopathy; ICD, International Classification of Diseases

Of the total 30,378 patients with HCM, 122 women experienced delivery after the HCM diagnosis. Patients with more than two pregnancies were classified as individual cases, resulting in 158 pregnancies. (Fig. 1) Baseline characteristics of all pregnancy cases are summarized in Table 1. The mean age of women was 34.1 ± 4.3 years old. Among them, cesarean section was performed in 78 (49.4%) pregnancies, and women who underwent cesarean section were older than those who opted for vaginal delivery. No women received an implantable cardioverter defibrillator (ICD) before delivery; however, septal myectomy had been performed in two women who delivered by cesarean section. There were no significant differences in the prevalence of hypertension, diabetes, stroke, AF, HF, and history of VT. No significant difference was observed in cardiovascular medications before pregnancy except RAS blockers. The period from the diagnosis of HCM to delivery was a median of 933 (interquartile range: 222–2088) days.

### Cardiovascular outcomes

**Fig. 2:**
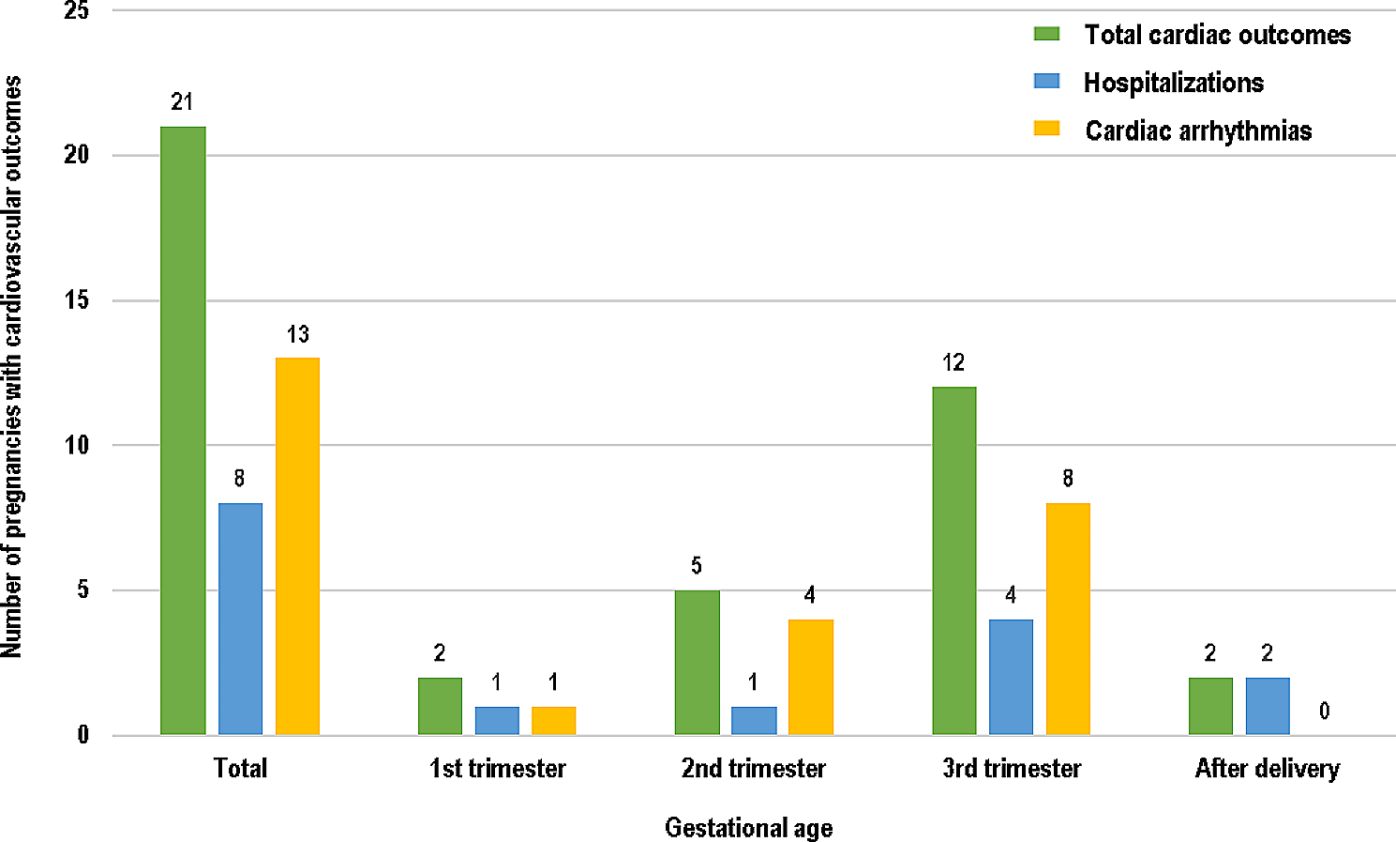
Number of cardiovascular outcomes in HCM during pregnancy Abbreviations: HCM, hypertrophic cardiomyopathy

Cardiovascular outcomes are summarized in Table 2 according to the trimester of the pregnancy. There was no maternal mortality in this data. During pregnancy and the first month postpartum, 21 cardiovascular events occurred. These events included two hospitalizations for HF, six hospitalizations for AF and 13 cases of new-onset arrhythmias. Among patients with new onset arrhythmia, eight new-onset AF and five new-onset VT were detected during pregnancy, but there was no thromboembolic event during pregnancy in patients with HCM. When evaluated based on the number of patients, a total of 14 (8.8%) individuals experienced cardiovascular events, with some patients experiencing two or more events in the whole period. Cardiac events occurred throughout pregnancy during the first, second, and a high frequency of events was noted during the third trimester (Fig. 2). There were two hospitalizations for cardiovascular outcomes after delivery, and both patients had undergone a cesarean section.

**Table 2:** Cardiovascular outcomes in patients with HCM during pregnancy

	Total	1st trimester	2nd trimester	3rd trimester	After delivery
**Hospitalization**	8 (5.1%)	1 (0.6%)	1 (0.6%)	4 (2.5%)	2 (1.3%)
Heart failure	2 (1.3%)	0 (0.0%)	0 (0.0%)	0 (0.0%)	2 (1.3%)
Atrial fibrillation	6 (3.8%)	1 (0.6%)	1 (0.6%)	4 (2.5%)	0 (0.0%)
**New onset arrhythmia**	13 (8.2%)	1 (0.6%)	4 (2.5%)	8 (5.1%)	0 (0.0%)
New onset AF	8 (5.1%)	1 (0.6%)	2 (1.3%)	5 (3.2%)	0 (0.0%)
New onset VT	5 (3.2%)	0 (0.0%)	2 (1.3%)	3 (1.9%)	0 (0.0%)
**Thromboembolic event**	0 (0.0%)	0 (0.0%)	0 (0.0%)	0 (0.0%)	0 (0.0%)

Abbreviations: HCM, hypertrophic cardiomyopathy; AF, atrial fibrillation; VT, ventricular tachycardia

### Obstetrical outcomes

Table 3 shows the obstetric outcomes for the total pregnancies. Regarding obstetrical complications, 23 (14.6%) patients with HCM experienced at least one complication, accounting for a total of 27 obstetric complications. Notably, most obstetrical complications developed after the second trimester and showed the highest rates in the third trimester. Preterm delivery occurred in seven of the 158 pregnancies (4.4%), and all preterm deliveries were performed in the third trimester. A cardiac event accompanied two cases of preterm delivery, and the period from cardiac event to delivery was 87 days and 164 days, respectively. In both cases, preterm delivery followed the diagnosis of new-onset atrial fibrillation.

**Table 3:** Obstetrical outcome in patients with HCM during pregnancy

	Total	1st trimester	2nd trimester	3rd trimester
Preeclampsia	2 (1.3%)	0 (0.0%)	1 (0.6%)	1 (0.6%)
Gestational hypertension	0 (0.0%)	0 (0.0%)	0 (0.0%)	0 (0.0%)
Gestational diabetes mellitus	10 (6.3%)	1 (0.6%)	2 (1.3%)	7 (4.4%)
Placenta previa	2 (1.3%)	0 (0.0%)	0 (0.6%)	2 (1.3%)
Obstructive labor	1 (0.6%)	0 (0.0%)	0 (0.0%)	1 (0.6%)
Abruptio placentae	0 (0.0%)	0 (0.0%)	0 (0.0%)	0 (0.0%)
Intrauterine growth retardation	3 (1.9%)	0 (0.0%)	0 (0.0%)	3 (1.9%)
Abortion	2 (1.3%)	2 (1.3%)	0 (0.0%)	0 (0.0%)
Preterm delivery	7 (4.4%)	0 (0.0%)	0 (0.0%)	7 (4.4%)

Abbreviations: HCM, hypertrophic cardiomyopathy

### Factors associated with cardiovascular outcomes or preterm delivery

As shown in the logistic regression analysis (Table 4), arrhythmias, including AF (odds ratio (OR): 7.44, 95% confidence interval (CI): 2.61–21.21, *P* < 0.001) and VT (OR: 31.61, 95% CI: 5.85–172.77, *P* < 0.001) diagnosed before pregnancy were significantly associated with cardiovascular outcomes or preterm delivery. Similar results were seen when only cardiovascular outcomes were analyzed separately (AF, OR: 10.67, 95% CI: 3.28–34.69, *P* < 0.001; VT, OR: 53.25, 95% CI: 9.24–306.93, *P* < 0.001, Supplementary Table 3). Older age, nulliparity, hypertension, diabetes, smoking, HF and surgical myectomy were not associated with adverse cardiovascular events and preterm delivery.

**Table 4:** Factors associated with cardiovascular outcomes or preterm delivery

	With cardiovascular outcomes or preterm delivery	Without cardiovascular outcomes or preterm delivery	OR	95% CI	*P*
	(*n* = 19, %)	(*n* = 139, %)			
Age > = 35	9 (47.4)	67 (48.2)	0.97	0.37–2.53	0.946
Age > = 40	2 (10.5)	13 (9.4)	1.14	0.24–5.50	0.87
Nulliparity	16 (84.2)	106 (76.3)	1.66	0.46–6.05	0.442
Hypertension	9 (47.4)	60 (43.2)	1.19	0.45–3.01	0.729
Diabetes mellitus	0 (0.0)	3 (2.2)	-	-	-
Atrial fibrillation	9 (47.4)	15 (10.8)	7.44	2.61–21.21	< 0.001
Heart failure	3 (15.8)	23 (16.5)	0.95	0.26–3.51	0.934
Ventricular tachycardia	6 (31.6)	2 (1.4)	31.61	5.85-172.77	< 0.001
Surgical myectomy	1 (5.3)	1 (0.7)	7.67	0.46-95.127.97	0.156
Smoking	1 (5.3)	10 (7.2)	0.72	0.09–5.94	0.758

* Cardiovascular outcomes: Maternal Death, admission for HF, AF, and Thromboembolic Events, New onset AF, VT

Abbreviations: HF, heart failure; AF, atrial fibrillation; AF, atrial fibrillation; VT, ventricular tachycardia

## Discussion

The present study investigated cardiovascular and obstetrical complications during pregnancy in patients with HCM. Patients diagnosed with HCM tolerated pregnancy well and did not have a high rate of cardiovascular or obstetrical complications. However, 8.8% of women experienced cardiovascular complications during pregnancy, resulting in hospitalization or treatment. These complications were more commonly observed in the second half of pregnancy. Among the underlying conditions, arrhythmias, such as AF and VT were related to adverse cardiovascular outcomes related to the pregnancy. Although most women with HCM can safely undergo pregnancy and childbirth, careful planning is necessary when arrhythmias are present.

### Prevalence of cardiovascular outcomes in pregnant women with HCM

HCM is one of the most prevalent hereditary heart diseases [14]; however, it is mainly diagnosed in individuals beyond middle age [2], resulting in a relatively low proportion of reproductive women with HCM. The physiological changes during pregnancy and childbirth impose additional hemodynamic challenges for individuals with HCM. Nevertheless, research in this area has been limited due to the small number of patients available for study. Moreover, most existing studies primarily focus on Western populations, with only a small-scale study involving approximately 30 Japanese participants [9]. Consequently, there is a significant dearth of studies examining the pregnancy-related impact of HCM on Asian populations. In this regard, our study is significant and relevant, because it represents the first comprehensive Asian study encompassing a substantial number of pregnant cases.

Previous research has consistently indicated a remarkably low occurrence of pregnancy-related mortality among patients with HCM [7–10]. This observation is supported by two relatively recent studies [9, 10], which reported no maternal death. Similarly, our study, which comprised 158 pregnancies, also showed no cases of maternal death. According to previous studies, the prevalence of cardiovascular complications in patients with HCM during pregnancy, such as HF and arrhythmia, has been reported to range from 15 to 45% [7, 9, 10, 15]. In our study, we observed cardiovascular issues in 8.8% of cases, which was slightly less frequent compared to previous findings. The feature of the Korean RID program, which comprehensively covers costs, may have influenced some of these differences. Some patients previously diagnosed with HCM may be admitted to the hospital based solely on the diagnosis of HCM when presenting with cardiovascular symptoms related to HCM. We identified eight cases in which patients were hospitalized with a diagnosis of HCM alongside concurrent cardiovascular symptoms such as dyspnea and chest pain without a diagnosis of HF or arrhythmias. Therefore, our findings may have underestimated the number of events compared to those of other studies.

In our study, the majority of cardiovascular events occurred during the third trimester of pregnancy. This pattern aligns with observations from two previous representative studies [9, 10]. The critical periods during pregnancy when patients are the most vulnerable to cardiac complications are typically the end of the first trimester, the second trimester at around 20 weeks’ gestation, at 32 to 34 weeks’ gestation, when blood volume is at its maximum, and during the peripartum period [16]. Given the consistent finding of a high frequency of events in the third trimester, both in similar studies and in our investigation, it can be concluded that pregnant women with HCM have an elevated risk of cardiovascular outcomes during this period. Hence, close observation is imperative during the third trimester to manage and mitigate potential complications.

### Arrhythmia in pregnant women with HCM

Our study highlighted arrhythmia as a prominent complication, consistent with the findings of a prior Japanese study [9] Notably, even within the general population, pregnancy is associated with a greater risk of arrhythmias, and patients with a history of arrhythmias are at significant risk of arrhythmia recurrence during pregnancy [17]. This heightened risk can be attributed to the physiologic changes of pregnancy, which include a decrease in parasympathetic nerves and an upsurge in sympathetic nerve activity. The increased sympathetic nerve response is intricately linked to automaticity, reentry, and triggered activity [17]. Hence, in individuals with HCM, who already exhibit a greater predisposition to arrhythmia compared to the general population, the likelihood of exacerbated arrhythmia during pregnancy is increased. Indeed, our study demonstrated that patients with underlying arrhythmias, such as AF or VT, were more prone to experience adverse cardiovascular outcomes. This emphasizes the importance of closely monitoring and providing appropriate care to patients with arrhythmias following pregnancy.

### Obstetric outcomes in pregnant women with HCM

In line with a prior retrospective cohort study that utilized large-scale NHIS data, which reported on obstetric complication rates in the general population, our study similarly identified obstetric complications such as preeclampsia, gestational hypertension, placenta previa, and abruptio placentae, occurring in approximately 1% of normal subjects. However, due to the limited sample size of patients in our study, making direct comparisons proves challenging. Nonetheless, we observed generally similar trends [18]. However, we did observe a higher incidence of gestational diabetes mellitus in the third trimester of pregnancy among patients with HCM. Currently, no existing research has explored the association between gestational diabetes mellitus and HCM. Further investigations in the future are required to confirm this relationship. Preterm delivery is a significant concern in pregnant women with HCM, which can lead to various complications. Our study identified seven preterm delivery cases and cardiovascular problems accompanied two cases. These findings align with the results of a Japanese study, wherein it was noted that approximately 30% of premature births were associated with underlying maternal heart issues [9]. This similarity further emphasizes the importance of monitoring and addressing the risk of preterm delivery in HCM pregnant women with cardiovascular complications.

### Delivery methods in pregnant women with HCM

In our study, approximately half of the pregnancy cases with HCM underwent a cesarean section for delivery, and no significant differences were observed in other baseline collected data. Notably, the cesarean section rate in the general population of Korea has shown an upward trend, increasing from 36.3 to 45% from 2006 to 2015 [19]. Considering our study’s inclusion of patients from 2006, it is reasonable to assume a higher prevalence of cesarean sections compared to the general population during that timeframe. While the literatures recommend vaginal delivery in cases of structural heart disease owing to the higher risks associated with cesarean section, such as bleeding, thromboembolic events, and infection, [20, 21] studies showed that cesarean sections have been more frequently performed in such cases [22]. Nevertheless, our findings indicate that there is no apparent need to prioritize cesarean section as the preferred method of delivery for patients with HCM.

### Study limitations

This study had several limitations. First, it is important to acknowledge that our study may have been susceptible to coding errors in diagnosing underlying diseases such as hypertension, diabetes, or HF. Since these diagnoses were based on diagnostic codes from the administrative database, there is a possibility of inaccuracies or misclassification in the coding process. This limitation should be considered when interpreting and generalizing the study results. Second, in patients with HCM, the echocardiography results are crucial in assessing the condition. However, we were unable to confirm these echocardiographic findings owing to the nature of the data source and study design. A previous study found that specific echocardiographic variables, such as left ventricular outflow tract obstruction and left ventricular ejection fraction, were not predictive indicators of major adverse cardiovascular events during pregnancy [9, 10]. Third, given the nature of the database, cardiac magnetic resonance or genetic test results could not be obtained. Fourth, considering the reimbursement characteristics of the Korea RID program, cardiovascular outcomes could be underestimated. Finally, our results were obtained only from Korean women; hence, its application to other ethnic groups may be limited.

## Conclusion

Based on the cohort of women diagnosed with HCM who underwent delivery in the current study, cardiovascular complication rate was low (8.8%) and obstetrical complications comparable to those observed in the general population except gestational diabetes mellitus. Consequently, it was noted that pregnancy and childbirth were generally well-tolerated by most women. However, for selected patients, particularly those with a history of arrhythmias, a planned delivery approach may be needed to mitigate the risks associated with cardiovascular outcomes.

### Electronic supplementary material

Below is the link to the electronic supplementary material.

Supplementary Material 1

## Data Availability

The datasets used and analyzed during the current study are available from the corresponding author on reasonable request.

## Abbreviations

AF: Atrial fibrillation
HCM: Hypertrophic cardiomyopathy
HF: Heart failure
ICD: Implantable cardioverter defibrillator
LMP: Last menstrual period
NIHS-HEALS: National Health Insurance Services-Health Screening
RAS: Renin-angiotensin-aldosterone system
RID: Rare intractable disease
VT: Ventricular tachycardia
