# Supplementary material for: Pregnancy related complications in women with hypertrophic cardiomyopathy: a nationwide population-based cohort study
Source: BMC Cardiovasc Disord. 2024 May 21;24:268. doi: 10.1186/s12872-024-03812-3 (PMC11106953; doi:10.1186/s12872-024-03812-3)
Supplement: Supplementary file 1 — Supplementary Material 1 [file 12872_2024_3812_MOESM1_ESM.docx]

**Supplementary Table 1. Definition of diseases**

| **Diagnosis** | **ICD-10-CM code and definition** |
| --- | --- |
| **Hypertrophic cardiomyopathy (HCM)** | Admission ≥1 or outpatient clinic ≥1  I421; obstructive hypertrophic cardiomyopathy or I422; other hypertrophic cardiomyopathy (nonobstructive hypertrophic cardiomyopathy); and the RID code of V127; cardiomyopathy-related diseases |
| ICD implantation | Z950 |
| Septal myectomy | O1825 |
| **Delivery** |  |
| Vaginal delivery | O82 (O820-P829), O842 |
| Cesarean section | O80 (O800-O809), O81(O810-O815), O83(O830-O839), O840, O841 |
| **Comorbidities** | Admission ≥1 or outpatient clinic ≥2 |
| Hypertension | I10-I13, I15; and minimum one prescription of anti-hypertensive drug (thiazide, loop diuretics, aldosterone antagonist, alpha-/beta-blocker, calcium-channel blocker, angiotensin-converting enzyme inhibitor, and angiotensin II receptor blocker). |
| Diabetes mellitus | E11-E14; and minimum one prescription of anti-diabetic drugs (sulfonylureas, metformin, meglitinides, thiazolidinediones, dipeptidyl peptidase-4 inhibitors, α-glucosidase inhibitors, SGLT2-inhibitor, GLP-1 agonist, and insulin). |
| Atrial fibrillation (AF) | I480-484, I489, I47.2, I49.0-49.4 |
| Heart Failure (HF) | I50 |
| Ventricular tachycardia (VT) | I47.2 |
| Stroke | I63 or I64 and HA441, 451, 461, 851; and admission with Brain CT  I63 or I64 and HE101, 201, 301, 401, 501; and admission with Brain MRI |
| **Cardiac Outcomes** |  |
| Hospitalization for HF | I50; and admission ≥1 |
| Hospitalization for AF | I480-484, I489, I47.2, I49.0-49.4; and admission≥1 |
| Hospitalization for VT | I47.2; and admission≥1 |
| Thromboembolic events | I63, I64, I26, O88.13; Admission ≥1 or outpatient clinic≥1 |
| **Obstetrical Outcomes** | Admission ≥1 or outpatient clinic ≥2 |
| Preeclampsia | O14 (O140-O149) |
| Gestational hypertension | O13 |
| Gestational diabetes mellitus | O244 |
| Placenta previa | O44 (O440-O441) |
| Obstructive labor | O64-O66 (O640-O669) |
| Abruptio placentae | O45 (O450-O459) |
| Intrauterine growth retardation | O365 |
| Abortion | O00-O08 |
| Preterm delivery | O601, O603 |

**Supplementary Table 2. Definition of pregnancy trimester**

| **Trimester** | **Period** |
| --- | --- |
| **Without preterm delivery code** |  |
| 1^st^ trimester | Between 39 and 27 weeks before delivery date (273-183 days before the delivery date) |
| 2^nd^ trimester | Between 26 and 14 weeks before delivery date (182-92 days before the delivery date) |
| 3^rd^ trimester | Between 13 weeks before delivery and the delivery date (91-0 days before the delivery date) |
| **With preterm delivery code** |  |
| 1^st^ trimester | Between 35 and 23 weeks before delivery date (245-155 days before the delivery date) |
| 2^nd^ trimester | Between 22 and 10 weeks before delivery date (154-64 days before the delivery date) |
| 3^rd^ trimester | Between 9 weeks before delivery and the delivery date (63-0 days before the delivery date) |

**Supplementary Table 3 Factors associated with cardiovascular outcomes**

|  | **With cardiovascular outcomes or preterm delivery** | **Without cardiovascular outcomes or preterm delivery** | **OR** | **95% CI** | **P-value** |
| --- | --- | --- | --- | --- | --- |
|  | **(n = 14, %)** | **(n = 144, %)** |  |  |  |
| Age >= 35 | 7 (50.0) | 69 (47.9) | 1.09 | 0.36-3.26 | 0.882 |
| Age >= 40 | 2 (14.3) | 13 (9.0) | 1.68 | 0.40-8.33 | 0.526 |
| Nulliparity | 12 (85.7) | 110 (76.4) | 1.86 | 0.40-8.70 | 0.434 |
| Hypertension | 7 (50.0) | 62 (43.1) | 1.32 | 0.44-3.97 | 0.618 |
| Diabetes mellitus | 0 (0.0) | 3 (2.1) | - | - | - |
| Atrial fibrillation | 8 (57.1) | 16 (11.1) | 10.67 | 3.28-34.69 | <0.001 |
| Heart failure | 3 (21.4) | 23 (16.0) | 1.44 | 0.37-5.55 | 0.601 |
| Ventricular tachycardia | 6 (42.9) | 2 (1.4) | 53.25 | 9.24-306.93 | <0.001 |
| Surgical myectomy | 1 (7.1) | 1 (0.7) | 11.00 | 0.65-186.33 | 0.100 |
| Smoking | 0 (0.0) | 11 (7.6) | - | - | - |

* Cardiovascular outcomes: Maternal Death, admission for HF, AF, and, Thromboembolic Events, New onset AF, VT

Abbreviations: HF, heart failure; AF, atrial fibrillation; AF, atrial fibrillation; VT, ventricular tachycardia
